# Phenotypic and comparative genomic characterization of a human biliary-derived *Kosakonia radicincitans* isolate

**DOI:** 10.3389/fmicb.2026.1885996

**Published:** 2026-06-25

**Authors:** Wanqiu Zhu, Guoxin Xu, Liping Gu, Xinfeng Geng, Yeye Dai, Fangyuan Qin, Xiaoxiao Pang, Yu Zheng, Long Chen, Xiaojue Zhu

**Affiliations:** Department of Clinical Laboratory, The First People’s Hospital of Zhangjiagang City, Zhangjiagang Hospital Affiliated to Soochow University, Zhangjiagang, China

**Keywords:** biliary, comparative genomics, Enterobacteriaceae, *Kosakonia radicincitan*s, MALDI-TOF MS

## Abstract

**Introduction:**

*Kosakonia radicincitans* is primarily recognized as a plant-associated and environmentally adapted member of Enterobacteriaceae, whereas recovery from human clinical specimens remains uncommon. Routine biochemical identification may misassign recently reclassified *Kosakonia* species to closely related *Enterobacter* taxa.

**Methods:**

We characterized strain ZJG61129, a *K. radicincitans* isolate recovered from bile during endoscopic retrograde cholangiopancreatography (ERCP) in a patient with choledocholithiasis. Colony morphology, VITEK 2 Compact identification, MALDI-TOF MS identification and antimicrobial susceptibility testing were performed. Whole-genome sequencing, average nucleotide identity (ANI), digital DNA–DNA hybridization (dDDH), 16S rRNA and core-genome phylogeny analyses, whole-genome comparison, pan-genome analysis, and resistance/virulence-associated gene screening were used for taxonomic confirmation and genomic characterization.

**Results:**

ZJG61129 formed smooth, moist colonies on blood agar and MacConkey agar. VITEK 2 Compact assigned the isolate to the *Enterobacter cloacae* complex with 92% confidence, whereas MALDI-TOF MS identified it as *K. radicincitans* with a score of 2.259. The genome consisted of a single circular chromosome of 5,510,622 bp with a GC content of approximately 54.0%. ANI and dDDH analyses confirmed assignment to *K. radicincitans*, and 16S rRNA together with core-genome phylogeny placed ZJG61129 within the *Kosakonia* lineage. Comparative genomics showed a conserved genomic backbone, substantial genomic plasticity, and no clear source-associated genomic structuring in the current dataset. ZJG61129 was susceptible to all antimicrobial agents tested. In silico resistance and virulence gene analyses did not indicate high-risk acquired resistance or a clearly high-virulence genotype.

**Discussion:**

ZJG61129 represents a human biliary-derived, environmental-like *K. radicincitans* isolate that may be misassigned by routine biochemical identification. Genome-based analysis is valuable for accurate recognition of uncommon, recently reclassified Enterobacteriaceae from biliary specimens.

## 1 Introduction

The genus *Kosakonia* comprises Gram-negative bacteria within the family Enterobacteriaceae and has been studied mainly in environmental and agricultural microbiology. Many *Kosakonia* strains have been isolated from plant-associated niches, including the rhizosphere, phyllosphere and endophytic compartments, where they may contribute to root colonization, nitrogen acquisition, nutrient mobilization and plant growth promotion. *Kosakonia radicincitans*, formerly known as *Enterobacter radicincitans*, is a representative plant growth-promoting species that has been evaluated as a beneficial inoculant in crop production systems (Silambarasan et al., 2022). Experimental studies have shown that *K*. *radicincitans* can colonize plant tissues and improve plant performance under both controlled and field conditions, supporting its role as an agriculturally beneficial and environmentally adapted bacterium rather than a typical human-associated microorganism (Quintas-Nunes et al., 2022; Shahid et al., 2022).

Although *Kosakonia* species are primarily regarded as plant- or environment-associated bacteria, sporadic human infections caused by members of this genus have been reported. *K. radicincitans* has been described as a rare cause of bloodstream infection, with isolates initially assigned to the *Enterobacter cloacae* complex by routine biochemical identification and later resolved using molecular or whole-genome approaches (Chen et al., 2023). Other members of the genus, particularly *K. cowanii*, have been implicated in acute cholecystitis, bacteremia and soft-tissue infections, often in patients with underlying conditions or environmental exposure (Yang et al., 2018; Wang et al., 2019; Legese et al., 2022; Barberis et al., 2026). These reports suggest that *Kosakonia* spp. may occasionally behave as opportunistic pathogens in specific clinical contexts, and that their occurrence in clinical microbiology may be underestimated because of limitations in conventional identification systems.

The clinical recognition of *Kosakonia* species is complicated by their taxonomic history and phenotypic similarity to closely related Enterobacteriaceae. *K. radicincitans* was transferred from *E. radicincitans* to the newly established genus *Kosakonia* by Brady et al. in 2013 (Kämpfer et al., 2005; Brady et al., 2013); therefore, its taxonomic predecessor is *E. radicincitans* rather than *E. cloacae*. This distinction is important because recently reclassified Enterobacteriaceae may be incompletely represented in routine biochemical databases. Consequently, *K. radicincitans* isolates may be assigned to the *E. cloacae* complex by automated biochemical systems, whereas MALDI-TOF MS and whole-genome sequencing provide higher discriminatory power when appropriate reference databases and genome-based metrics are available (Werinder et al., 2021). Average nucleotide identity and core-genome phylogeny are now widely used for species-level confirmation and for resolving relationships among closely related Enterobacteriaceae (Liu et al., 2025).

The biliary tract represents a relevant but underexplored context for human-associated environmental Enterobacteriaceae. Increasing evidence indicates that bile and biliary stone-associated microbial communities may contain diverse bacteria, including members of the Enterobacteriaceae. In patients with biliary stones or ductal dilatation, altered bile flow and intraductal stones may facilitate bacterial retention, attachment or persistence within the biliary system (Liu et al., 2015; Sistrunk et al., 2016). In this context, the recovery of an environmentally associated genus such as *Kosakonia* from bile raises the possibility that the biliary tract may serve as a transient ecological niche or potential reservoir for atypical Enterobacteriaceae (Liu et al., 2015; Berinson et al., 2020). Such organisms may remain clinically silent in some individuals but could become clinically relevant under conditions such as biliary obstruction, inflammation, endoscopic or surgical manipulation, or host immune compromise.

In the present study, we characterized strain ZJG61129, a *K. radicincitans* isolate recovered from bile during endoscopic retrograde cholangiopancreatography (ERCP) in a patient with choledocholithiasis. Routine identification using the VITEK 2 Compact system assigned the isolate to the *E. cloacae* complex, whereas MALDI-TOF MS identified it as *K. radicincitans*. To resolve this discrepancy and define the biological features of the isolate, we performed phenotypic characterization, antimicrobial susceptibility testing, whole-genome sequencing, average nucleotide identity analysis, core-genome SNP phylogeny, BRIG-based comparative genomics, and in silico screening of antimicrobial resistance and virulence-associated genes. By comparing ZJG61129 with publicly available *K*. *radicincitans* genomes, other *Kosakonia* species and *E. cloacae* complex genomes, we aimed to clarify its taxonomic placement, assess the limitations of routine biochemical identification, and determine whether a human biliary-derived isolate exhibits genomic features distinct from environmental *K*. *radicincitans* strains.

## 2 Materials and methods

### 2.1 Sample collection and bacterial culture

Bile was collected aseptically during Endoscopic Retrograde Cholangiopancreatography (ERCP) stone extraction and submitted for routine bacterial culture. The sample was inoculated onto blood agar and MacConkey agar plates and incubated at 35 °C for 24 h under aerobic conditions. Colony morphology was recorded.

### 2.2 Bacterial identification

Routine bacterial identification was performed using the VITEK 2 Compact system (bioMérieux, Marcy-l’Étoile, France) according to the manufacturer’s instructions. MALDI-TOF MS identification was performed using the MALDI Biotyper system (Bruker Daltonics, Bremen, Germany) with the manufacturer’s reference database. Identification scores were interpreted according to the manufacturer’s criteria. Because VITEK 2 Compact and MALDI-TOF MS yielded discordant identification results, whole-genome sequencing was performed for definitive taxonomic confirmation.

### 2.3 Antimicrobial susceptibility testing

Antimicrobial susceptibility testing was performed using VITEK 2 AST-N335/XN04 panels according to the 2025 Clinical and Laboratory Standards Institute guidelines (CLSI M100, 2025). All experimental procedures were conducted with three independent biological replicates, prepared on separate dates. *Escherichia coli* ATCC 25922 was used for quality control.

### 2.4 Whole-genome sequencing, assembly and annotation

Genomic DNA was extracted from an overnight culture of ZJG61129 using Solarbio Bacterial Genomic DNA Extraction Kit (Beijing, China). Whole-genome sequencing was performed using Illumina NovaSeq X Plus platform (Illumina, San Diego, CA, USA) and PacBio Sequel system (Pacific Biosciences, Menlo Park, CA, USA). Raw Illumina reads were quality-filtered using TrimGalore v0.5.0 and FastQC. Genome assemblies were *de novo* generated using SPAdes v3.15.3, with hybrid assemblies combining Illumina short reads and PacBio long reads where available. Genome circularization was assessed using Circlator and manually inspected based on terminal overlap and read support. Genome annotation was conducted using RAST and Prokka 1.10. Antimicrobial resistance genes were screened using CARD DB v4.0.1 and virulence-associated genes were screened using VFDB. Assembly quality was assessed based on contig number, genome size, N50, GC content, sequencing depth, genome coverage, BUSCO completeness and CheckM completeness/contamination estimates. Plasmid replicons were screened using PlasmidFinder.

### 2.5 Phylogenetic tree and comparative genomic analysis

For comparative genomic analysis, 16 publicly available *K. radicincitans* genomes, including the reference strain DSM 107547, were retrieved from the NCBI database. In addition, the 11 closest other *Kosakonia* genomes and the 14 closest *E. cloacae* complex genomes to ZJG61129 were selected and included to evaluate interspecies relationships. Metadata including strain name, species, isolation source, country and accession number were compiled in Supplementary Table S1. Core-genome SNPs were identified using kSNP v3.0. The optimal k-mer size was determined using Kchooser, and SNPs present in the core genome were retained for phylogenetic reconstruction. A maximum-likelihood phylogenetic tree was constructed using IQ-TREE v2.4.0. The best-fit nucleotide substitution model was selected using ModelFinder, and branch support was assessed using 1,000 ultrafast bootstrap replicates. The tree was visualized and annotated using iTOL v7. ZJG61129 was analyzed together with representative *K. radicincitans*, other *Kosakonia* species and *E. cloacae* complex genomes. The 16S rRNA sequences were extracted from genome assemblies using Barrnap v0.9. Among the 42 genomes screened, complete or high-quality 16S rRNA sequences were recovered from 36 genomes and aligned using MAFFT v7. Phylogenetic reconstruction was performed using IQ-TREE v2.4.0 with 1,000 ultrafast bootstrap replicates.

Average nucleotide identity (ANI) was calculated between ZJG61129 and the selected reference genomes using pyANI.^1^ ANI values ≥95% were considered to indicate species-level relatedness. ANI results were visualized as a heatmap using R. Digital DNA–DNA hybridization (dDDH) values were calculated using the Type (Strain) Genome Server (TYGS). A dDDH value of 70% was considered the generally accepted species boundary.

Comparative genomic analysis of chromosomal architectures was conducted using BRIG v0.95 to visualize sequence homology and structural variations against the reference genome. Pan-genome analysis was performed using Panaroo v1.1.2. Core and accessory gene families were identified, and pan-genome openness was evaluated by fitting Heap’s law. Accessory genes associated with the currently available human-derived genomes were identified from the pan-genome matrix and functionally annotated using KEGG and the NCBI non-redundant (NR) database.

## 3 Results

### 3.1 Clinical background, isolation, and initial identification of strain ZJG61129

**Figure 1:**
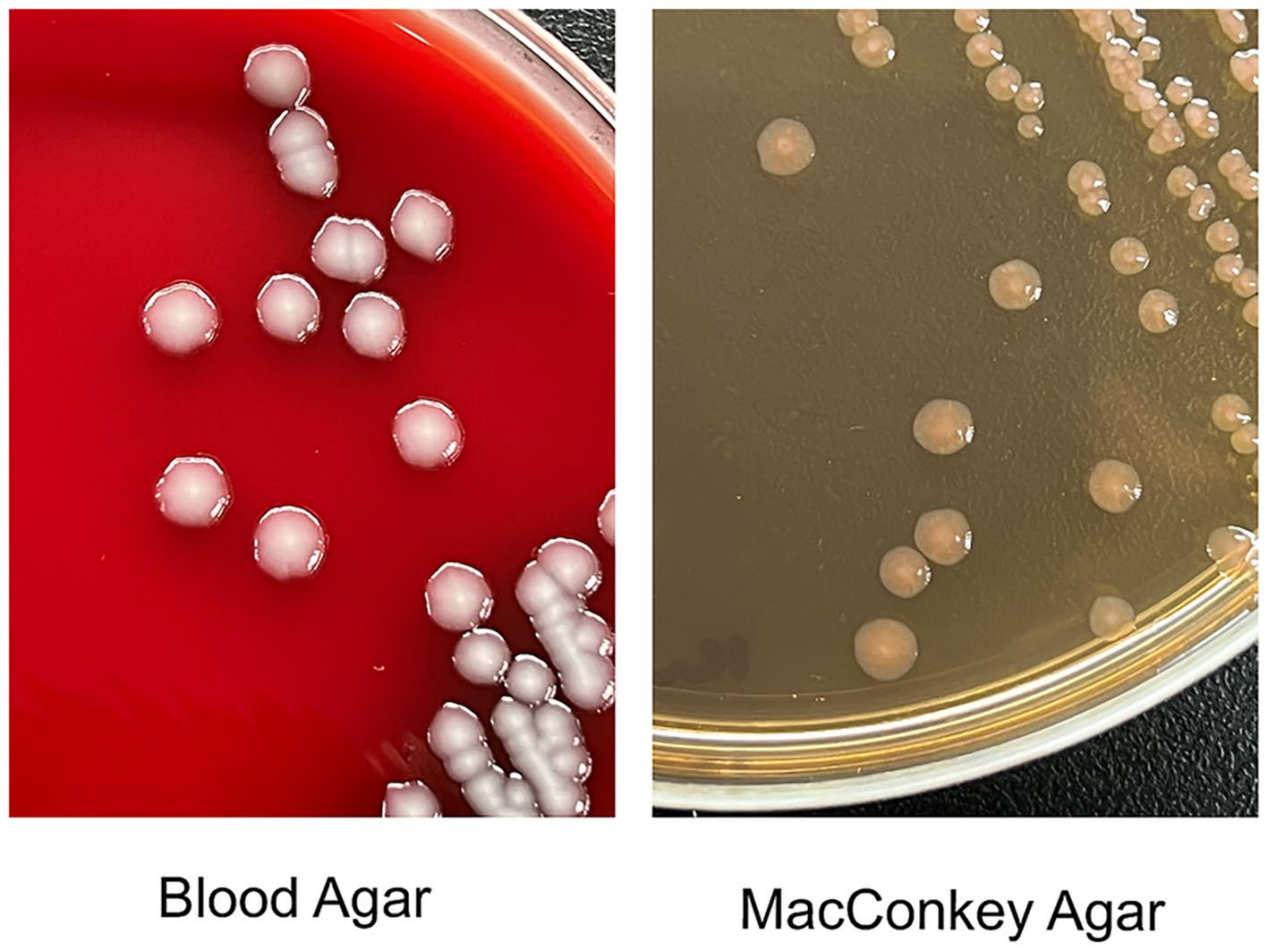
Colony morphology of *Kosakonia radicincitans* ZJG61129 on blood agar and MacConkey agar. The bile sample was cultured aerobically on blood agar and MacConkey agar at 37 °C for 24 h. Colonies were smooth, moist and convex; they appeared milky-white to grayish-white on blood agar and pale pink to light pink on MacConkey agar.

Strain ZJG61129 was recovered from a bile sample collected during ERCP stone extraction from a 59-year-old male patient with common bile duct stones and choledochal dilation. On admission, the patient showed no remarkable clinical signs of infection, with a white blood cell count of 3.51 × 10^9^/L and a neutrophil percentage of 48.6%. Following postoperative supportive therapy, the patient recovered uneventfully and was discharged 1 week after the procedure. After 24 h of incubation, ZJG61129 formed circular, smooth, moist, convex colonies with entire margins on both blood agar and MacConkey agar. Colonies on blood agar were milky-white to grayish-white and showed no obvious hemolysis, whereas those on MacConkey agar were pale pink to light pink (Figure 1). Routine identification using the VITEK 2 Compact system assigned ZJG61129 to the *E. cloacae* complex with a confidence of 92%, whereas MALDI-TOF MS identified the isolate as *K. radicincitans* with a score of 2.259, exceeding the manufacturer’s threshold for reliable species-level identification. This discordance prompted whole-genome-based taxonomic confirmation.

### 3.2 General genomic features of ZJG61129

Whole-genome sequencing of ZJG61129 using a hybrid short-read and long-read strategy generated a high-quality single-contig genome assembly. The genome was assembled as a single circular chromosome of 5,510,622 bp, with no ambiguous bases and a GC content of approximately 54.0%. The assembly had an N50 of 5,510,622 bp, a sequencing depth of 199.84 × and 100% genome coverage. Genome quality assessment showed 124 complete BUSCOs, corresponding to 100% BUSCO completeness, and CheckM estimated 99.57% completeness with 1.71% contamination. No plasmid sequence or plasmid replicon was detected, indicating that ZJG61129 likely lacks plasmids. Genome annotation predicted 5,041 protein-coding sequences, 22 rRNA genes, 82 tRNA genes, 11 ncRNA genes and 65 pseudogenes (Supplementary Figure S1). Together, these results indicate that ZJG61129 was assembled into a highly contiguous and complete genome suitable for downstream comparative genomic analyses.

### 3.3 Genome-based taxonomic assignment of ZJG61129

**Figure 2:**
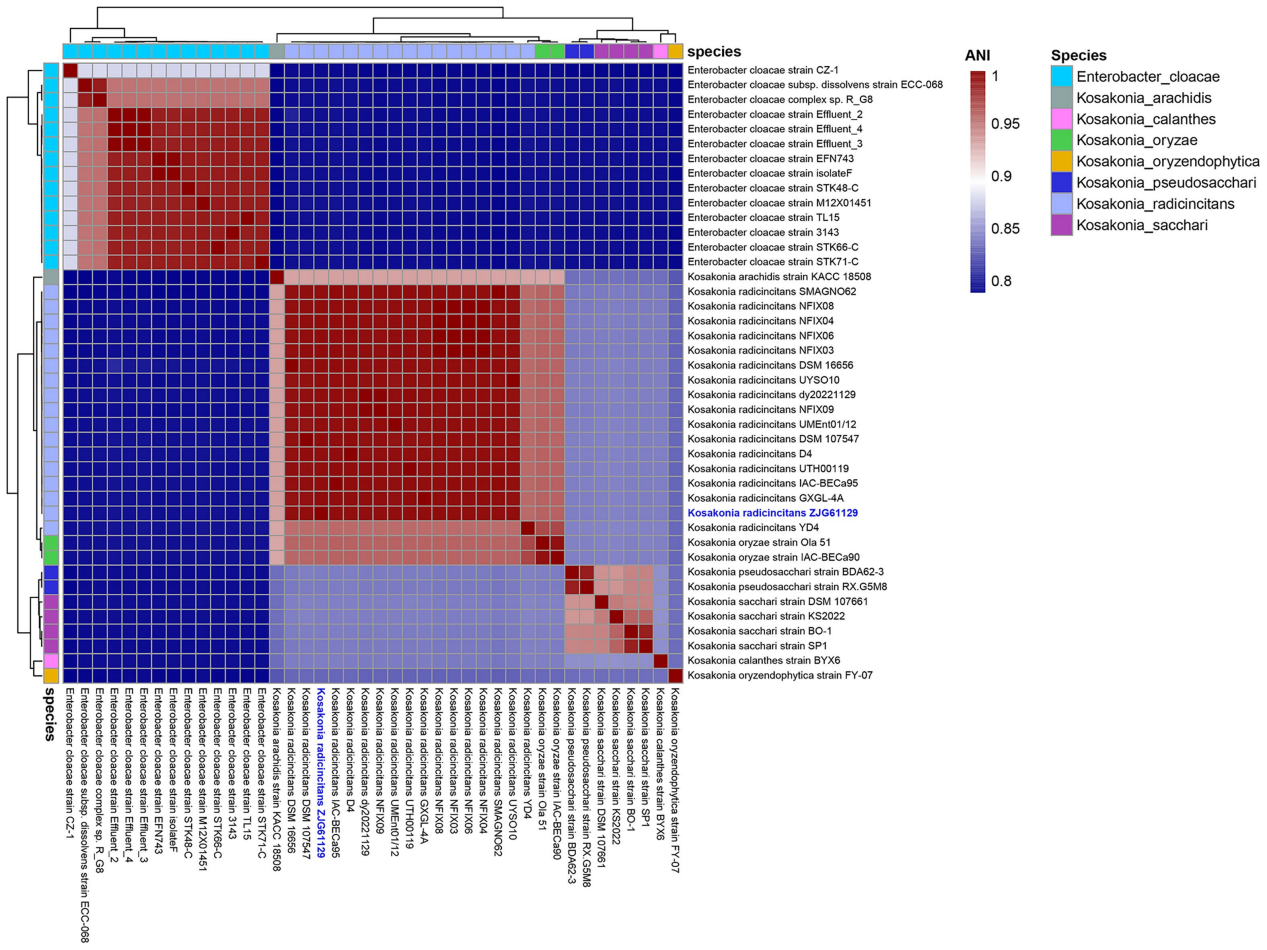
Average nucleotide identity analysis of ZJG61129 and reference genomes. ANI heatmap showing pairwise genomic relatedness between ZJG61129, 16 publicly available *K. radicincitans* genomes, representative genomes from other *Kosakonia* species and 14 *E. cloacae* complex genomes. ZJG61129 shared 95.54–99.42% ANI with public *K. radicincitans* genomes; the lowest value was observed with *K. radicincitans* YD4 (95.54%), whereas ANI values with all other *K. radicincitans* genomes exceeded 99%. Relatively high ANI values were observed with two *K. oryzae* genomes (95.89%) and *K. arachidis* strain KACC 18508 (93.28%), while ANI values with the 14 *E. cloacae* complex genomes were all below 80%.

Genome-based analyses were performed to determine the taxonomic position of ZJG61129. ANI values between ZJG61129 and 17 publicly available *K. radicincitans* genomes ranged from 95.54 to 99.42%. The lowest ANI value was observed with *K. radicincitans* YD4 (95.54%), whereas ANI values with the remaining *K. radicincitans* genomes, including the reference strain DSM 107547, exceeded 99%, supporting the classification of ZJG61129 as *K*. *radicincitans*. Among other *Kosakonia* species, the highest ANI values were observed with two *K. oryzae* genomes (95.89% for both), whereas ANI values with *K*. *arachidis* and other *Kosakonia* species ranged from 83.18 to 93.28%. In contrast, ANI values between ZJG61129 and the 14 *E. cloacae* complex genomes were all below 80%, clearly excluding affiliation with the *E. cloacae* complex (Figure 2).

To further support species assignment, digital DNA–DNA hybridization (dDDH) analysis was performed and yielded results consistent with the ANI analysis (Supplementary Figure S2). ZJG61129 showed dDDH values above the generally accepted species threshold with most *K. radicincitans* genomes, whereas lower values were observed for other *Kosakonia* species and the *E. cloacae* complex. The two *K. oryzae* genomes that showed relatively high ANI values also exhibited comparatively high dDDH values, reflecting the close genomic relationship between these closely related *Kosakonia* taxa.

A 16S rRNA-based phylogeny was constructed using 36 genomes from which complete or high-quality 16S rRNA sequences could be recovered; six genomes were excluded because no suitable 16S rRNA sequence could be extracted from the assemblies. In the resulting tree, ZJG61129 grouped within the *Kosakonia* lineage, although closely related *Kosakonia* species were not clearly resolved, reflecting the limited discriminatory power of 16S rRNA sequences at the species level (Supplementary Figure S3).

**Figure 3:**
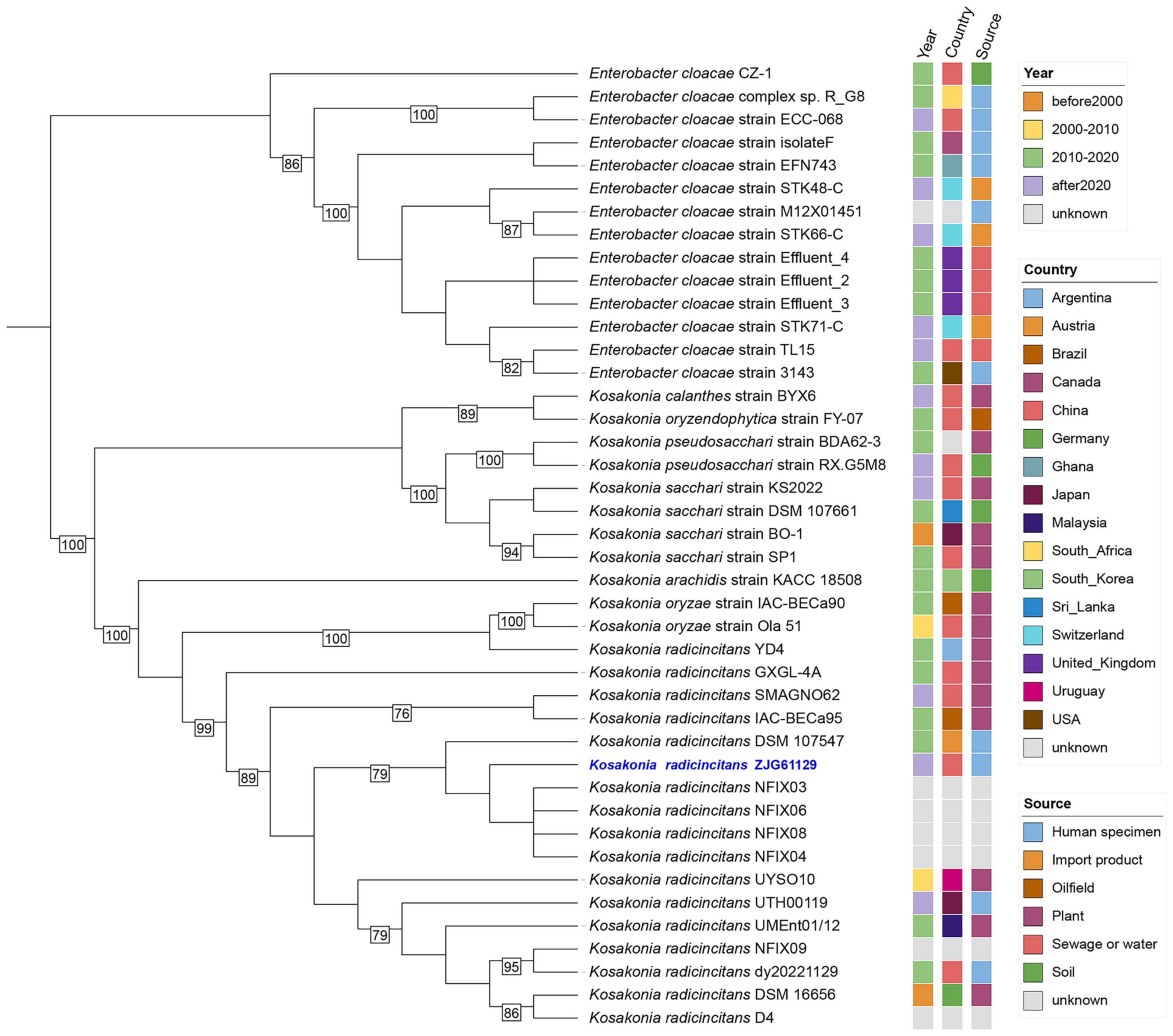
Core-genome SNP phylogeny of ZJG61129 and reference genomes. Maximum-likelihood phylogenetic tree based on core-genome SNPs showing the relationship of ZJG61129 to public *K. radicincitans* genomes, representative genomes from other *Kosakonia* species and *Enterobacter cloacae* complex genomes. ZJG61129 clustered within the main *K. radicincitans* clade, whereas the genome annotated as *K. radicincitans* YD4 grouped closer to *K. oryzae*. All included *Kosakonia* genomes were clearly separated from the *E. cloacae* complex.

Core-genome SNP phylogeny provided higher taxonomic resolution. Sixteen *K*. *radicincitans* genomes, including ZJG61129 and the reference strain DSM 107547, formed a well-supported clade, whereas the genome annotated as *K. radicincitans* YD4 clustered more closely with *K. oryzae*. At a broader taxonomic scale, all included *Kosakonia* genomes formed a distinct lineage that was clearly separated from the *E. cloacae* complex genomes (Figure 3). Together, these genome-based analyses confirmed the classification of ZJG61129 as *K. radicincitans* and supported the MALDI-TOF MS identification.

### 3.4 Comparative genomic and pan-genome analyses of ZJG61129

**Figure 4:**
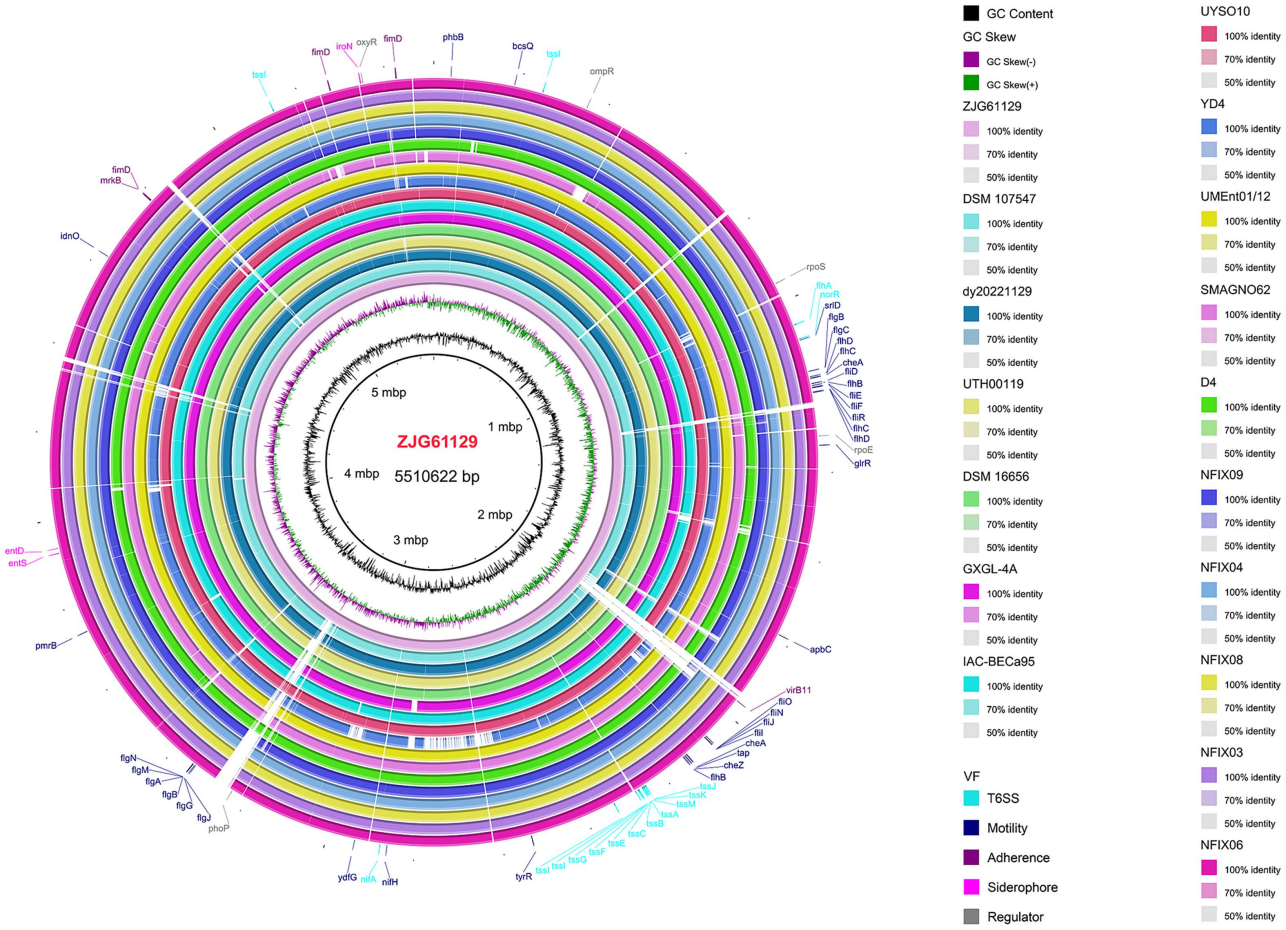
BRIG-based comparative genomic analysis of ZJG61129 and public *K. radicincitans* genomes. Circular genome comparison showing sequence similarity between ZJG61129 and 16 publicly available *K. radicincitans* genomes. Concentric rings represent individual genomes aligned against the reference genome. ZJG61129 shared a highly conserved genomic backbone with public *K. radicincitans* genomes, without large ZJG61129-specific regions or broad genomic regions uniquely associated with human-derived isolates.

BRIG comparison demonstrated that ZJG61129 shared a highly conserved genomic backbone with the available *K*. *radicincitans* genomes (Figure 4). No large genomic island or major structural region unique to ZJG61129 was identified. Likewise, no broad genomic region was consistently shared only among the currently available human-derived isolates or absent from environmental and plant-associated genomes.

Pan-genome analysis of the 17 available *K. radicincitans* genomes identified 8,208 gene families, comprising 4,175 core genes and 4,033 accessory genes. The pan-genome accumulation curve remained unsaturated, and the fitted *γ* value of 0.548 indicated an open pan-genome, suggesting ongoing acquisition of accessory genes and considerable genomic plasticity (Supplementary Figure S4).

Comparative analysis of the currently available human- and plant-derived genomes identified accessory gene families associated with the human-derived isolates (Supplementary Figure S5). Functional annotation indicated that these genes were mainly related to mobile genetic elements, type IV secretion systems, iron acquisition and recombination-associated functions. Representative annotations included type IV secretion system components and yersiniabactin-associated iron acquisition genes (Supplementary Table S4). However, most of these genes were strain-specific rather than universally shared among all human-derived isolates and therefore were interpreted as accessory genomic features rather than evidence of a distinct human-adapted lineage.

### 3.5 Antimicrobial susceptibility and in silico resistome/virulome profiles

Antimicrobial susceptibility testing showed that ZJG61129 was susceptible to all antimicrobial agents tested (Table 1), with no multidrug-resistant phenotype observed. The isolate remained susceptible to *β*-lactam/β-lactamase inhibitor combinations, cephalosporins, aztreonam, carbapenems, fluoroquinolones, aminoglycosides, tetracycline-class agents, polymyxin B and trimethoprim/sulfamethoxazole (Table 1). CARD screening identified 26 strict resistance-associated hits, mainly corresponding to intrinsic efflux systems and regulatory components, including representative high-identity hits such as *oqxB*, *baeR*, *marA*, *mdtB*, *mdtC*, *emrR* and *msbA*. No common high-risk acquired resistance genes, including ESBL genes, carbapenemases, plasmid-mediated quinolone resistance genes or *mcr* family genes, were detected (Supplementary Table S2).

**Table 1:** Antimicrobial susceptibility profiles of ZJG61129.

Antimicrobial agent	MIC (μg/mL)	Interpretation
Amoxicillin/Clavulanic acid	≤2	S
Piperacillin	8	S
Cephalothin	≤2	S
Cefuroxime	2	S
Cefuroxime axetil	2	S
Cefotetan	≤4	S
Cefpodoxime	≤0.25	S
Cefotaxime	≤1	S
Ceftizoxime	≤1	S
Aztreonam	≤1	S
Doripenem	≤0.12	S
Meropenem	≤0.25	S
Nalidixic acid	≤2	S
Moxifloxacin	≤0.25	S
Norfloxacin	≤0.5	S
Tetracycline	≤1	S
Tigecycline	≤0.5	S
Piperacillin/Tazobactam	≤4	S
Ceftazidime	≤0.12	S
Cefoperazone/Sulbactam	≤8	S
Cefepime	≤0.12	S
Imipenem	≤0.25	S
Amikacin	≤2	S
Tobramycin	≤1	S
Ciprofloxacin	≤0.25	S
Levofloxacin	≤0.12	S
Doxycycline	≤0.5	S
Minocycline	≤1	S
Polymyxin B	≤0.5	S
Trimethoprim/Sulfamethoxazole	≤20	S

VFanalyzer identified 82 virulence factor-associated hits in ZJG61129, mainly involving secretion system-associated components, adherence, iron uptake, motility and surface structure-related genes. The most prominent categories included flagellar genes, T6SS-associated components, pilus-related genes, heme/siderophore-associated iron acquisition genes, and LPS/O-antigen or capsule-associated loci. However, the curated output did not support the presence of a complete classical toxin locus, complete type III secretion system or major pathogenicity island (Supplementary Table S3). These results suggest that the virulence-associated repertoire of ZJG61129 mainly reflects basic colonization and environmental fitness traits rather than a clearly high-virulence phenotype. Genes associated with iron acquisition, oxidative stress responses and membrane stress-related functions were also identified, including siderophore- and heme-related homologues and transport-associated systems. These genes may contribute to bacterial fitness under stressful or nutrient-limited conditions but are widely conserved among environmental Enterobacteriaceae.

## 4 Discussion

In this study, we characterized ZJG61129, a *Kosakonia radicincitans* isolate recovered from bile during ERCP in a patient with choledocholithiasis. Using phenotypic testing, MALDI-TOF MS, antimicrobial susceptibility testing, whole-genome sequencing and comparative genomics, we confirmed its taxonomic identity and evaluated its relationship to public *K. radicincitans*, other *Kosakonia* species and *E. cloacae* complex genomes. Our findings provide additional evidence that environmental-like *Kosakonia* strains may occasionally be recovered from human biliary specimens (Liu et al., 2015; Berinson et al., 2020).

A key finding was the discordance between routine biochemical identification and MALDI-TOF MS. ZJG61129 was assigned to the *E. cloacae* complex by VITEK 2 Compact but identified as *K. radicincitans* by MALDI-TOF MS. Genome-based analyses confirmed the MALDI-TOF MS result. This discrepancy likely reflects the limitations of phenotype-based identification systems for recently reclassified Enterobacteriaceae. Importantly, *K. radicincitans* was formerly classified as *Enterobacter radicincitans* (Kämpfer et al., 2005), not *E. cloacae*, before being transferred to the genus *Kosakonia* (Brady et al., 2013). Therefore, assignment to the *E. cloacae* complex should be interpreted as a biochemical misassignment rather than true taxonomic affiliation.

ANI and core-genome SNP phylogeny provided robust support for the classification of ZJG61129 as *K. radicincitans*. The isolate showed >99% ANI with most public *K. radicincitans* genomes and was clearly separated from the *E. cloacae* complex (Figure 2). The outlying position of the genome annotated as *K. radicincitans* YD4, which grouped closer to *K. oryzae*, is consistent with previous genome-based evidence showing that YD4 is more closely related to *K. oryzae* than to the *K. radicincitans* type lineage (Li et al., 2017). In addition, two genomes annotated as *K. oryzae* showed ANI values exceeding 95% relative to ZJG61129. Although their dDDH values remained below the accepted species threshold, these findings suggest a close evolutionary relationship and raise the possibility of taxonomic ambiguity or misclassification among closely related *Kosakonia* genomes. The 16S rRNA analysis supported placement of ZJG61129 within *Kosakonia*, but its limited resolution among closely related species further justified the use of genome-wide metrics, including ANI, dDDH and core-genome SNP phylogeny, for species-level identification.

Comparative genomic analysis showed that ZJG61129 was broadly similar to other *K. radicincitans* genomes. BRIG analysis revealed a conserved genomic backbone without large ZJG61129-specific regions or broad genomic regions uniquely associated with human-derived isolates (Figure 4). The open pan-genome suggests substantial genomic plasticity within *K. radicincitans*, which may facilitate adaptation to diverse environmental and host-associated niches. Accessory genes associated with the currently available human-derived genomes included functions related to iron acquisition, type IV secretion systems and mobile genetic elements. Iron acquisition and membrane stress-related functions may contribute to bacterial survival under stressful host-associated conditions, including bile. However, these functions are broadly conserved among environmental Enterobacteriaceae and should not be interpreted as evidence of specific adaptation to the human biliary tract. Because most accessory genes were strain-specific, they should not be interpreted as evidence of stable human adaptation. Given the limited number of human-derived genomes, our data do not allow firm conclusions regarding human-specific adaptation, and the present dataset did not show clear source-associated genomic structuring.

The antimicrobial susceptibility, CARD and VFanalyzer results support a low-risk phenotype. ZJG61129 was susceptible to all tested antimicrobial agents, and CARD hits mainly reflected intrinsic efflux or regulatory systems rather than high-risk acquired resistance determinants (Supplementary Table S2). VFanalyzer annotations were largely associated with motility, adherence, nutrient acquisition and surface structures, without evidence of a complete classical toxin locus, complete type III secretion system or major pathogenicity island (Supplementary Table S3). Combined with the absence of overt infection in the patient, these findings suggest that ZJG61129 is better interpreted as an environmental-like biliary isolate with low pathogenic potential rather than a definitive biliary pathogen.

The recovery of ZJG61129 from bile raises the possibility that the biliary tract may serve as a transient niche for environmental-like Enterobacteriaceae. Increasing evidence suggests that the biliary tract harbors a resident or transient microbiota, particularly under conditions such as biliary obstruction or gallstone disease, and that bile and stone-associated microbial communities can include members of the Enterobacteriaceae (Gunn et al., 2014; Bednarsch et al., 2021). Altered bile flow, stone formation and endoscopic manipulation may facilitate transient bacterial persistence or biofilm formation within the biliary tract. However, our data do not demonstrate long-term colonization or a causal role in disease. This study is also limited by the analysis of a single newly isolated strain, the absence of functional assays for bile tolerance, adhesion, invasion, biofilm formation or virulence, and the limited number of public human-derived *K. radicincitans* genomes. In addition, CARD and VFanalyzer annotations are sequence similarity-based and require cautious interpretation.

In conclusion, ZJG61129 represents a human biliary-derived *K. radicincitans* isolate that was assigned to the *E. cloacae* complex by routine biochemical testing but confirmed as *K. radicincitans* by MALDI-TOF MS and genome-based analyses. The isolate was genomically close to environmental and plant-associated *K. radicincitans* strains, showed no clear source-associated genomic structuring in the current dataset, and exhibited a susceptible antimicrobial phenotype without high-risk acquired resistance determinants. These findings emphasize the value of genome-based identification for recently reclassified Enterobacteriaceae and suggest that the biliary tract may provide a transient niche for environmental-like *K. radicincitans* strains.

## Data Availability

The genome assembly of Kosakonia radicincitans ZJG61129 has been deposited in NCBI under BioProject PRJNA1467270. The assembly is available under GenBank accession GCA_057732915.1 and RefSeq accession GCF_057732915.1.

## Footnotes

1
https://github.com/widdowquinn/pyani

## References

[ref1] Barberis C. Haim M. S. Zomero P. Traglia G. Ellis A. Cittadini R. . (2026). Two cases of posttraumatic Kosakonia infection, Argentina, 2023. Emerg. Infect. Dis. 32, 459–462. doi: 10.3201/eid3203.251714, 41863765 PMC13016020

[ref2] Bednarsch J. Czigany Z. Heij L. R. Luedde T. van Dam R. Lang S. A. . (2021). Bacterial bile duct colonization in perihilar cholangiocarcinoma and its clinical significance. Sci. Rep. 11:2926. doi: 10.1038/s41598-021-82378-y, 33536484 PMC7858613

[ref3] Berinson B. Bellon E. Christner M. Both A. Aepfelbacher M. Rohde H. (2020). Identification of Kosakonia cowanii as a rare cause of acute cholecystitis: case report and review of the literature. BMC Infect. Dis. 20:366. doi: 10.1186/s12879-020-05084-6, 32448208 PMC7245821

[ref4] Brady C. Cleenwerck I. Venter S. Coutinho T. De Vos P. (2013). Taxonomic evaluation of the genus Enterobacter based on multilocus sequence analysis (MLSA). Syst. Appl. Microbiol. 36, 309–319. doi: 10.1016/j.syapm.2013.03.005, 23632228

[ref5] Chen Z. Tang L. Yuan C. E J. Wang D. Liu X. . (2023). *Kosakonia radicincitans* with hypervirulent lON genes causes human bloodstream infections. Future Microbiol. 18, 317–322. doi: 10.2217/fmb-2022-0190, 37140352

[ref6] Gunn J. S. Marshall J. M. Baker S. Dongol S. Charles R. C. Ryan E. T. (2014). Salmonella chronic carriage: epidemiology, diagnosis, and gallbladder persistence. Trends Microbiol. 22, 648–655. doi: 10.1016/j.tim.2014.06.007, 25065707 PMC4252485

[ref7] Kämpfer P. Ruppel S. Remus R. (2005). *Enterobacter radicincitans* sp. nov., a plant growth promoting species of the family Enterobacteriaceae. Syst. Appl. Microbiol. 28, 213–221. doi: 10.1016/j.syapm.2004.12.007, 15900968

[ref8] Legese M. H. Asrat D. Swedberg G. Hasan B. Mekasha A. Getahun T. . (2022). Sepsis: emerging pathogens and antimicrobial resistance in Ethiopian referral hospitals. Antimicrob. Resist. Infect. Control 11:83. doi: 10.1186/s13756-022-01122-x, 35698179 PMC9195281

[ref9] Li Y. Li S. Chen M. Peng G. Tan Z. An Q. (2017). Complete genome sequence of Kosakonia oryzae type strain Ola 51T. Stand. Genomic Sci. 12:28. doi: 10.1186/s40793-017-0240-8, 28428833 PMC5392936

[ref10] Liu Q. Shen H. Wei M. Chen X. Gu L. Zhu W. (2025). Global phylogeography and antibiotic resistance characteristics of Morganella: An epidemiological, spatial, comparative genomic study. Drug Resist. Updat. 78:101180. doi: 10.1016/j.drup.2024.101180, 39657433

[ref11] Liu J. Yan Q. Luo F. Shang D. Wu D. Zhang H. . (2015). Acute cholecystitis associated with infection of Enterobacteriaceae from gut microbiota. Clin. Microbiol. Infect. 21, 851.e1–851.e9. doi: 10.1016/j.cmi.2015.05.017, 26025761

[ref12] Quintas-Nunes F. Rossi M. J. Nascimento F. X. (2022). Genomic insights into the plant-associated lifestyle of Kosakonia radicincitans MUSA4, a diazotrophic plant-growth-promoting bacterium. Syst. Appl. Microbiol. 45:126303. doi: 10.1016/j.syapm.2022.126303, 35149280

[ref13] Shahid M. Al-Khattaf F. S. Danish M. Zeyad M. T. Atef Hatamleh A. Mohamed A. . (2022). PGPR Kosakonia Radicincitans KR-17 increases the salt tolerance of radish by regulating ion-homeostasis, photosynthetic molecules, redox potential, and stressor metabolites. Front. Plant Sci. 13:919696. doi: 10.3389/fpls.2022.919696, 35979076 PMC9376370

[ref14] Silambarasan S. Logeswari P. Sivaramakrishnan R. Cornejo P. Sipahutar M. K. Pugazhendhi A. (2022). Amelioration of aluminum phytotoxicity in *Solanum lycopersicum* by co-inoculation of plant growth promoting Kosakonia radicincitans strain CABV2 and *Streptomyces corchorusii* strain CASL5. Sci. Total Environ. 832:154935. doi: 10.1016/j.scitotenv.2022.154935, 35395302

[ref15] Sistrunk J. R. Nickerson K. P. Chanin R. B. Rasko D. A. Faherty C. S. (2016). Survival of the fittest: how bacterial pathogens utilize bile to enhance infection. Clin. Microbiol. Rev. 29, 819–836. doi: 10.1128/CMR.00031-16, 27464994 PMC5010752

[ref16] Wang C. Wu W. Wei L. Feng Y. Kang M. Xie Y. . (2019). Kosakonia quasisacchari sp. nov. recovered from human wound secretion in China. Int. J. Syst. Evol. Microbiol. 69, 3155–3160. doi: 10.1099/ijsem.0.003606, 31355737

[ref17] Werinder A. Aspán A. Söderlund R. Backhans A. Sjölund M. Guss B. . (2021). Whole-genome sequencing evaluation of MALDI-TOF MS as a species identification tool for *Streptococcus suis*. J. Clin. Microbiol. 59:e0129721. doi: 10.1128/JCM.01297-21, 34469186 PMC8525557

[ref18] Yang X. J. Wang S. Cao J. M. Hou J. H. (2018). Complete genome sequence of human pathogen Kosakonia cowanii type strain 888-76(T). Braz. J. Microbiol. 49, 16–17. doi: 10.1016/j.bjm.2017.03.010, 28774637 PMC5790570

