# Supplementary material for: Phenotypic and comparative genomic characterization of a human biliary-derived Kosakonia radicincitans isolate
Source: Front Microbiol. 2026 Jun 25;17:1885996. doi: 10.3389/fmicb.2026.1885996 (PMC13346056; doi:10.3389/fmicb.2026.1885996)
Supplement: Supplementary file 2 [file Table_2.DOCX]

**Supplementary Table S2. Curated summary of CARD-based antimicrobial resistance annotations in ZJG61129.**

| **Category** | **Representative CARD hits** | **Result in ZJG61129** | **Interpretation** |
| --- | --- | --- | --- |
| Overall CARD hits | 26 strict hits | Present | Mainly intrinsic/resistance-associated homologues |
| Efflux systems/regulators | oqxB, baeR, marA, mdtB, mdtC, emrR, msbA | Present | Common intrinsic or regulatory resistance-associated background |
| ESBL genes | bla*_CTX-M_*, bla*_SHV_*, bla*_TEM_* | Not detected | Consistent with cephalosporin susceptibility |
| Carbapenemases | bla_NDM_, bla_KPC_, bla_VIM_, bla_IMP_, bla_OXA-48_-like | Not detected | Consistent with carbapenem susceptibility |
| Plasmid-mediated quinolone resistance | qnr, aac(6')-Ib-cr | Not detected | Consistent with fluoroquinolone susceptibility |
| Colistin resistance | mcr family | Not detected | Consistent with polymyxin B susceptibility |
| Low-identity homologues | e.g., vanG-, adeF-, PBP3-, fosA-like hits | Present in raw output if applicable | Not interpreted as functional determinants |
