# Supplementary material for: Phenotypic and comparative genomic characterization of a human biliary-derived Kosakonia radicincitans isolate
Source: Front Microbiol. 2026 Jun 25;17:1885996. doi: 10.3389/fmicb.2026.1885996 (PMC13346056; doi:10.3389/fmicb.2026.1885996)
Supplement: Supplementary file 3 [file Table_3.DOCX]

**Supplementary Table S3. Curated summary of VFanalyzer-based virulence factor annotations in ZJG61129.**

| **VF category** | **Representative genes/loci** | **Interpretation** |
| --- | --- | --- |
| Flagella/motility | flgB, flgC, flgF, flgG, flhB, flhC, flhD, fliA, fliC, fliD, fliF, fliG, fliM, fliN, fliP, fliQ, fliR, fliS | Motility-associated traits |
| T6SS-associated components | SCI-I, AAI/SCI-II, H-T6SS, T6SS-III components | Component-level matches; completeness not experimentally confirmed |
| Adherence | ecpA-ecpE, hcpA-hcpC, afaB, afaC, fimD | Potential colonization-associated structures |
| Iron acquisition | chuS, chuU, iroE, enterobactin-related homologues, hemB, pvdH-like | Iron acquisition and nutrient scavenging functions potentially associated with survival under iron-limited conditions |
| Surface structures | capsule-, LPS/LOS-, O-antigen-related genes, galU, uge, wbaP/rfbP | Common Gram-negative surface-associated features, envelope integrity and environmental adaptation |
| Efflux/biofilm-related | acrB, adeG-like | May contribute to membrane stress tolerance and environmental fitness |
| Toxin/protease-like | hlyB, pla-like | Single homologues; no complete toxin locus supported |
