# Supplementary material for: Phenotypic and comparative genomic characterization of a human biliary-derived Kosakonia radicincitans isolate
Source: Front Microbiol. 2026 Jun 25;17:1885996. doi: 10.3389/fmicb.2026.1885996 (PMC13346056; doi:10.3389/fmicb.2026.1885996)
Supplement: Supplementary file 4 [file Table_4.DOCX]

**Supplementary Table S4. Representative accessory genes identified by comparative pan-genome analysis of the currently available human-derived Kosakonia radicincitans genomes.**

| **Functional category** | **Representative genes/loci** | **Annotation source** | **Predicted function** | **Potential biological relevance** |
| --- | --- | --- | --- | --- |
| Type IV secretion system (T4SS) | *virB1*, *virB2*, *virB4*, *virB5*, *virB6*, *virB8*, *virB9*, *virB10*, *virB11*, *traW*, *trbJ*, *trbL* | KEGG | Type IV secretion system | May facilitate horizontal gene transfer and ecological adaptation |
| Iron acquisition / siderophore-associated system | *fyuA*, *irp1*, *irp2*, *irp3*, *irp4*, *irp5*, *irtA*, *mbtI* | KEGG | Yersiniabactin-associated iron acquisition | May contribute to persistence under iron-limited host-associated environments |
| Mobile genetic elements | Integrase, transposase, recombinase-related proteins | NR | Genome rearrangement and gene mobilization | May promote accessory gene acquisition and genome plasticity |
| DNA recombination and repair | *recF*, *lexA*, *ssb*, *topB* | NR | DNA repair and homologous recombination | May facilitate genome maintenance and adaptive evolution |
| Restriction-modification system | *hsdR*, *hsdM*, *hsdS* | NR | Type I restriction-modification system | May contribute to defense against foreign DNA and genome stability |
