# Supplementary material for: Phenotypic and comparative genomic characterization of a human biliary-derived Kosakonia radicincitans isolate
Source: Front Microbiol. 2026 Jun 25;17:1885996. doi: 10.3389/fmicb.2026.1885996 (PMC13346056; doi:10.3389/fmicb.2026.1885996)
Supplement: Supplementary file 5 [file Data_Sheet_1.PDF]

Figure S1

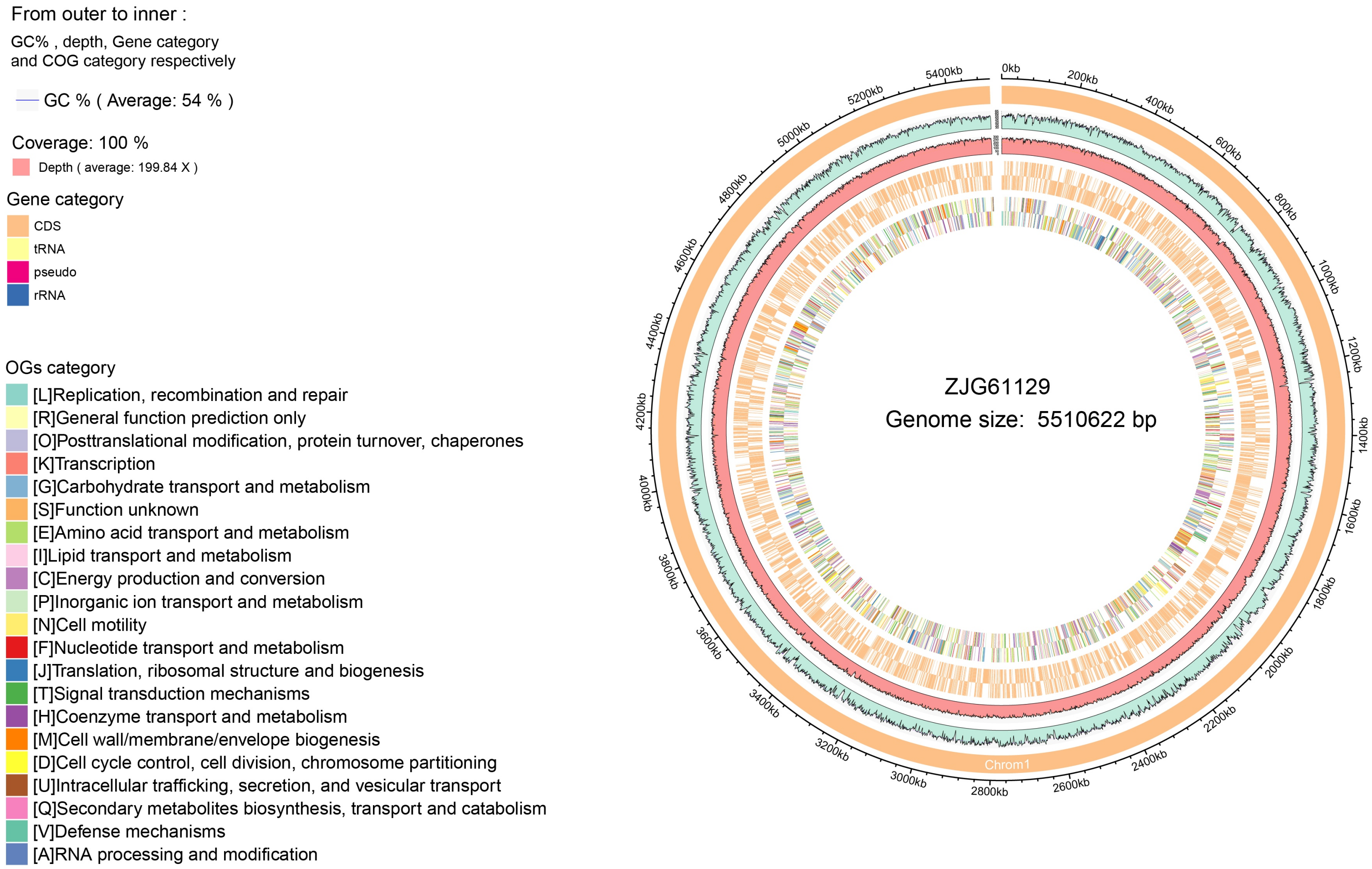

Figure S1. Circular genome map of *Kosakonia radicincitans* ZJG61129. Tracks from outer to inner represent GC content, sequencing depth, gene categories and COG functional categories. The genome consisted of a single contig of 5,510,622 bp, with an average GC content of 54.0%, 100% coverage and an average sequencing depth of 199.84×.

Figure S2

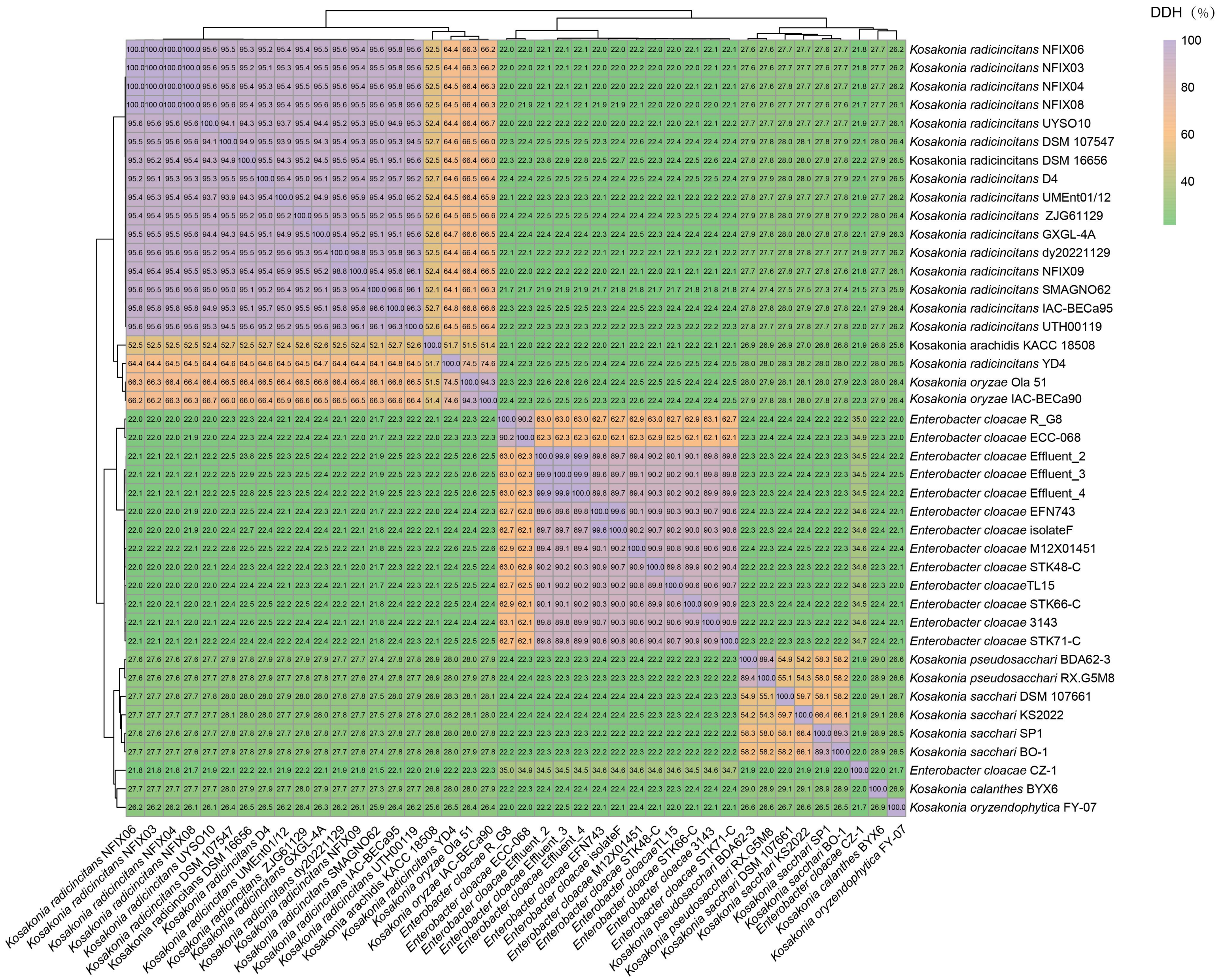

Figure S2. Pairwise digital DNA-DNA hybridization (dDDH) heatmap of ZJG61129 and representative *Kosakonia* and *Enterobacter cloacae* complex genomes. Pairwise dDDH values were calculated using TYGS and visualized by hierarchical clustering. The commonly accepted species threshold for dDDH is 70%.

Figure S3

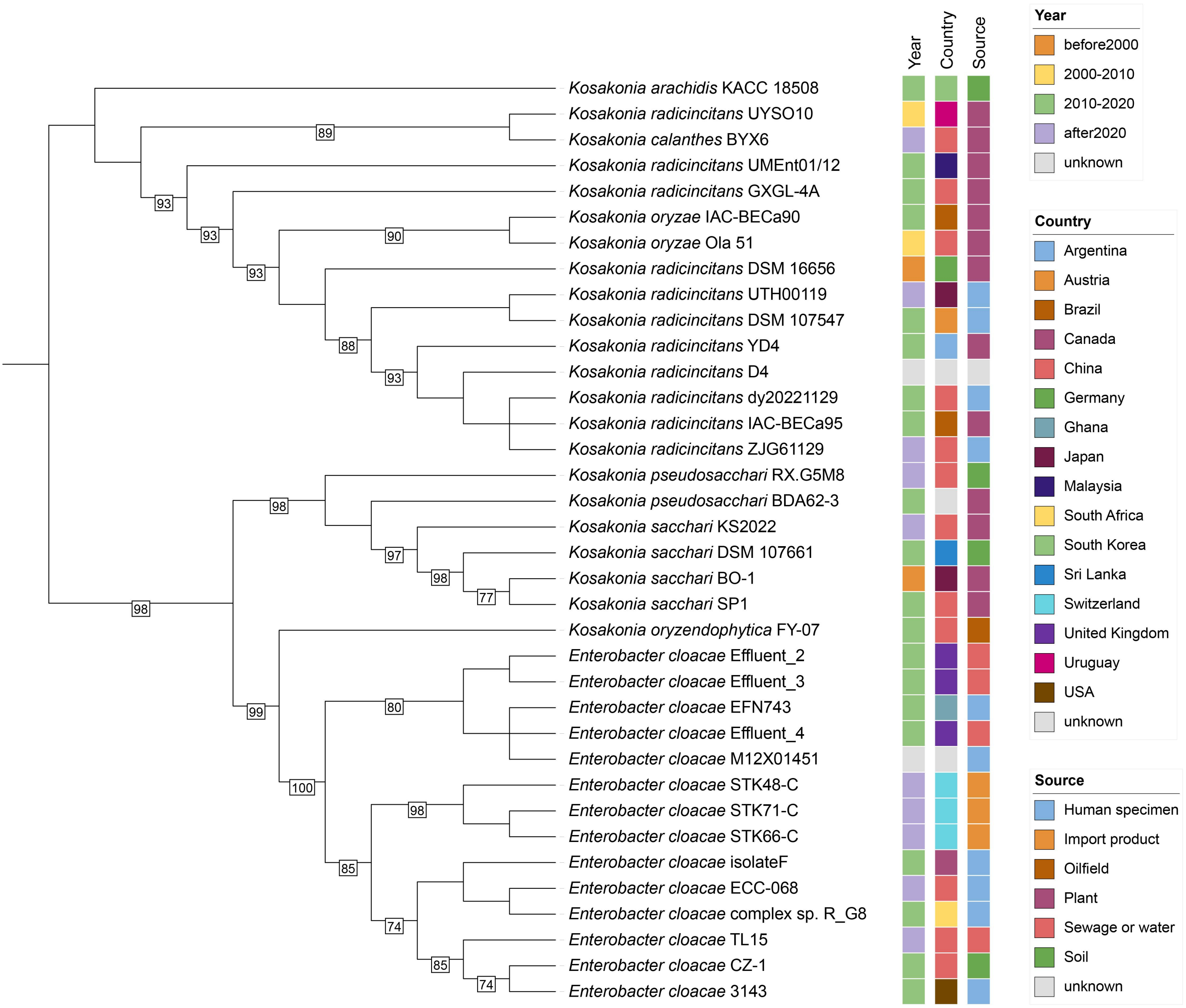

Figure S3. 16S rRNA-based phylogeny of ZJG61129 and representative reference genomes. The phylogenetic tree was constructed using 16S rRNA sequences extracted from 36 genomes. Among the 42 genomes screened, six were excluded because no complete or high-quality 16S rRNA sequence could be extracted from the assemblies. ZJG61129 grouped within the *Kosakonia* lineage, but the 16S rRNA tree showed limited resolution among closely related *Kosakonia* species.

Figure S4

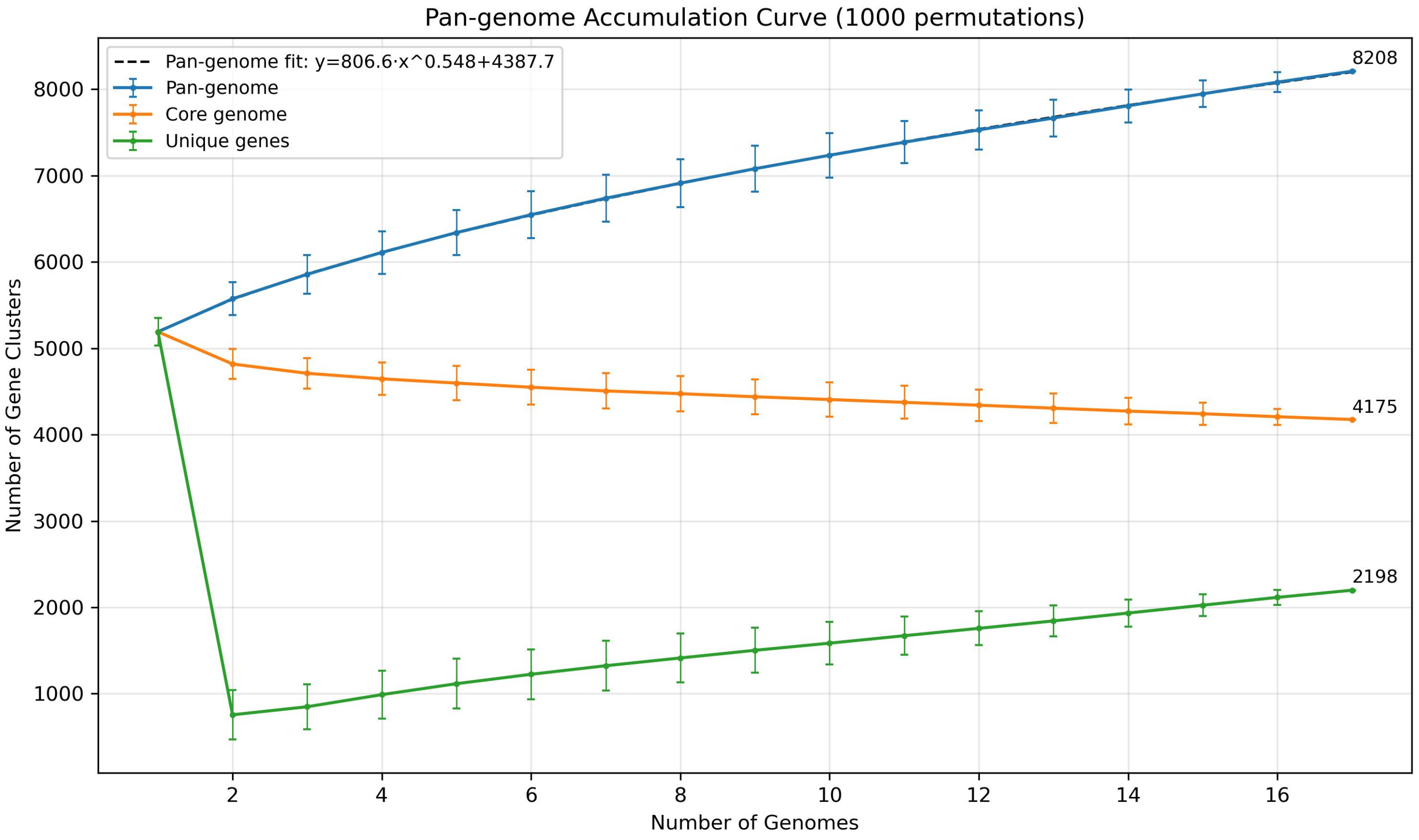

Figure S4. Pan-genome analysis of 17 publicly available *K. radicincitans genomes*. The accumulation curves of the pan-genome and core genome are shown. The unsaturated pan-genome curve and the fitted γ value indicate that *K. radicincitans* possesses an open pan-genome.

Figure S5

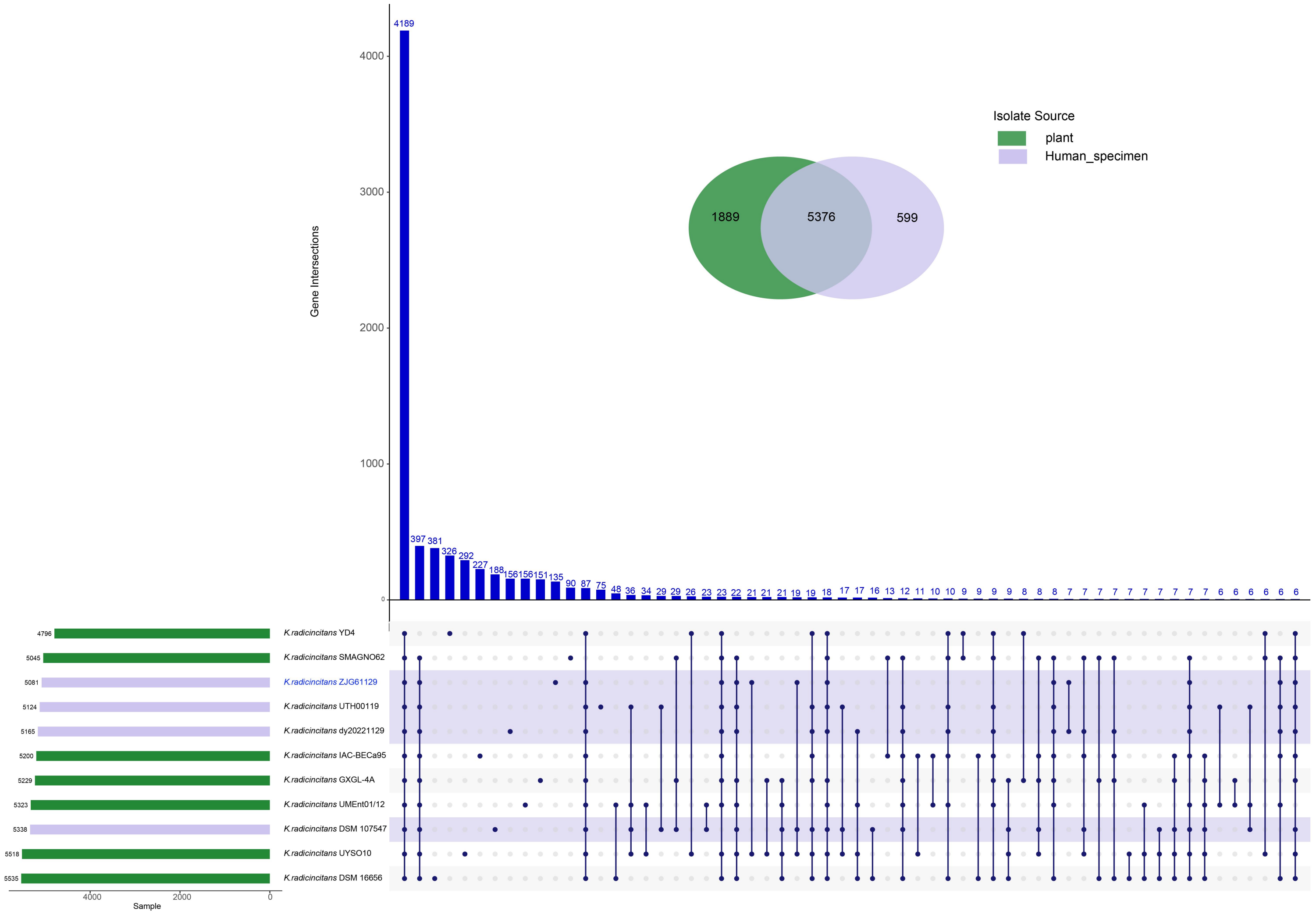

Figure S5. Distribution of accessory gene families among human- and plant-derived *K. radicincitans* genomes. Accessory gene families associated with the currently available human-derived genomes were identified by comparative pan-genome analysis.
